# EEG-Based Biometrics: Challenges And Applications

**DOI:** 10.1155/2018/5483921

**Published:** 2018-06-07

**Authors:** Victor Hugo C. de Albuquerque, Robertas Damaševičius, João Manuel R. S. Tavares, Plácido R. Pinheiro

**Affiliations:** ^1^Programa de Pós Graduação em Informática Aplicada, Laboratório de Bioinformática, Universidade de Fortaleza, Fortaleza, CE, Brazil; ^2^Department of Software Engineering, Kaunas University of Technology, Kaunas, Lithuania; ^3^Instituto de Ciência e Inovação em Engenharia Mecânica e Engenharia Industrial, Departamento de Engenharia Mecânica, Faculdade de Engenharia, Universidade do Porto, Porto, Portugal

## 1. Introduction

Biometrics is aimed at recognizing individuals based on physical, physiological, or behavioural characteristics of a human body such as fingerprint, gait, voice, iris, and gaze. Currently, the state-of-the-art methods for biometric authentication are being incorporated in various access control and personal identity management applications. While the hand-based biometrics (including fingerprint) have been the most often used technology so far, there is growing evidence that electroencephalogram (EEG) signals collected during a perception or mental task can be used for reliable person recognition. However, the domain of EEG-based biometry still faces the problems of improving the accuracy, robustness, security, privacy, and ergonomics of the EEG-based biometric systems and substantial efforts are needed towards developing efficient sets of stimuli (visual or auditory) that can be used of person identification in Brain-Computer Interface (BCI) systems and applications.

There are still many challenging problems involved in improving the accuracy, efficiency, and usability of EEG-based biometric systems and problems related to designing, developing, and deploying new security-related BCI applications, for example, for personal authentication on mobile devices, augmented and virtual reality, headsets, and Internet.

This special issue is aimed to introduce the recent advances of EEG-based biometrics and addresses the challenges in developing the EEG-based biometry systems for various practical applications, while proposing new ideas and directions for future development, such as data preprocessing, feature extraction, recognition, and matching; signal processing and machine learning techniques; EEG biometric based passwords and encryption; cancellable EEG biometrics; multimodal (EEG, EMG, ECG, and other biosignals) biometrics; pattern recognition techniques; protocols, standards, and interfaces; security and privacy; information fusion for biometrics involving EEG data, virtual environment applications, stimuli sets, and passive BCI technology.

## 2. Computational Intelligence Techniques

This special issue 6 published original works selected from 13 submitted articles, addressing new trends in the field from several novel methods and techniques used in different applications, for instance, A. Gumaei et al. proposed a novel method based on autoencoder and regularized extreme learning machine to make recognition faster by reducing the number of palmprint features without degrading the accuracy of the classifier, in which the results were high compared to the recent studies and proved the robustness and efficiency of the proposed technique.

R. Damaševičius et al. presented a cryptographic authentication approach (using a dataset of electroencephalography data collected from 42 subjects) based on the discrete logarithm problem and Bose-Chaudhuri-Hocquenghem codes for security analysis, showing that the proposed biometric user authentication method presented satisfactory results.

K. A. A. Cruz et al. evaluated n-iterative exponential forgetting factor for EEG signals parameter estimation, showing the effectiveness of technique thought of the comparison of three forms of iterative-recursive uses of the Exponential Forgetting Factor combined with a linear function to identify a synthetic stochastic signal.

M. Fazlyyyakhmatov et al. investigated a cortical activity during the cognitive task consisted of binocular viewing of a false image, which is observed when the eyes are refocused out of the random-dot stereogram plane (3D phenomenon) and concluded that during stereo perception of the false image the power of alpha-band activity decreased in the left parietal area and bilaterally in frontal areas of the cortex, while activity in beta-1, beta-2, and delta frequency bands remained unchanged.

N. Yu et al. evaluated a new sparse coding algorithm using p-norm optimization in single-trial evoked potentials (EPs) estimating, in which we can track the underlying EPs corrupted by *α*-stable distribution noise, trial-by-trial, without the need to estimate the *α* value. Simulations and experiments on human visual evoked potentials and event-related potentials are carried out to examine the performance of the proposed approach, concluding that the proposed method is effective in estimating single-trial EPs under impulsive noise environment.

R. D. de C. Silva et al. proposed a novel tool based on augmented reality to reduce the stigma related to schizophrenia, simulating the psychotic symptoms typical of schizophrenia and simulating sense perception changes in order to create an immersive experience capable of generating pathological experiences of a patient with schizophrenia, presenting a robust tool, quite realistic and, thus, very promising to reduce stigma associated with schizophrenia by instilling in the observer a greater comprehension of any person during a schizophrenic outbreak, whether a patient or a family member.

